# Effect of including oilseed grains in bovine diets on fatty acid profile, lipid stability, and sensory aspects of burgers

**DOI:** 10.3389/fvets.2022.923937

**Published:** 2022-07-21

**Authors:** Thais Rayane Rios Brito, Heitor Henrique Costa Valeriano, Luís Carlos Vinhas Ítavo, Marjorie Toledo Duarte, Marília Williani Filgueira Pereira, Samara Miyaki Corrêa, Luciana Miyagusku, Thiago Luís Alves Campos de Araújo, Camila Celeste Brandão Ferreira Ítavo, Rodrigo da Costa Gomes, Marina de Nadai Bonin Gomes

**Affiliations:** ^1^College of Veterinary Medicine and Animal Science, Federal University of Mato Grosso do Sul, Campo Grande, Brazil; ^2^Department of Food Technology and Public Health, Federal University of Mato Grosso do Sul, Campo Grande, Brazil; ^3^Animal Science Department, Federal University of Ceara, Fortaleza, Brazil; ^4^Embrapa Beef Cattle, Campo Grande, Brazil

**Keywords:** burgers quality, fatty acids, lipid oxidation, meat products, sensory analysis

## Abstract

The aim of this study was to evaluate the effect of including different oilseed grains in the diets of cattle on the qualitative and sensory characteristics and fatty acid profile of burger over a storage period of up to 120 days. The soybean diet increased 30% of ether extract in burgers when compared to the control diet. The inclusion of oilseeds in the bovine diet did not change the *n*-6/*n*-3 and hypocholesterolemic/hypercholesterolemic ratio, as well as the cholesterol levels in the burgers. The smallest flavor and aroma note scores were attributed to burgers produced with meat of bovine fed with cottonseed (4.35 and 4.67, respectively). The sunflower diet resulted in smaller lipid oxidation (1.03 mg/kg). The storage period increased lipid oxidation (0.43 and 1.97 mg/kg of malonaldehyde at 0 and 120 days, respectively). The inclusion of oilseeds in the diet of cattle does not change the ratios of fatty acids in burgers, which are important to human health. It is recommended to use soybean and sunflower grains in cattle diets to improve the sensory quality of burgers. A 30-day storage period is recommended to maintain the flavor and juiciness of beef burgers.

## Introduction

Beef has a high nutritional value, containing proteins, vitamins, minerals, fatty acids, and essential nutrients. However, due to the high amounts of saturated fatty acids and low amounts of monounsaturated and polyunsaturated fatty acids (PUFAs), it is associated with the development of cardiovascular diseases (1, 2). Foods rich in PUFAs, have been shown to have beneficial health effects by reducing levels of “bad” cholesterol (low-density lipoproteins; LDL) and platelet agglomeration, and increasing the levels of “good” cholesterol (high-density lipoproteins; HDL) in the blood (1, 3). Lipids are important constituents of meat which increase the deposition efficiency and the quality of deposited fat (4), contributing to better sensory characteristics such as flavor, juiciness and fatty acid profile (5).

The use of oilseed grains in ruminant nutrition can change the fatty acid profile of the meat by increasing the levels of PUFAs (6, 7). PUFAs are stored in the cell membranes of the muscle fiber and are more susceptible to oxidation, due to their double-bonded structure (8). Lipid oxidation is the main cause of fatty acid deterioration (9) as it is a spontaneous and inevitable process and one of the main problems faced by the industry, changing the meat sensory characteristics as color, odor, and so, the acceptance by the consumers and shelf life of the products (10, 11).

This problem could be more evident in meat processed products, as burgers, obtained from ground meat, molded, and submitted to an appropriate technological process, leading to the cell membranes disruption and exposition of lipid fractions to free radicals that produce volatile compounds and cause the oxidation of the product (12). In addition, the freezing process used for the long time storage of these products, at the same time that stabilizes the microorganism, can accentuate the process of lipid oxidation by the action of oxygen on the lipids (13).

In this way, to determine the effects of the composition of bovine diets on the increase of the unsaturated fatty acid (UFA) content in the meat, and its influence on technological and sensory characteristics of the product is essential for the beef cattle production chain (7, 14, 15). The aim of this study is to evaluate the effect of including oilseed grains in bovine diets on lipid stability, qualitative and sensory characteristics, and the fatty acid profile of burgers subjected to different storage periods.

## Materials and Methods

### Animals and slaughter

For the burger processing, meat samples from 24 Nellore steers, feedlot finished for 112 days were used. The animals received a diet of 40% corn silage as a roughage source and 60% of concentrate composed with no oilseeds grains added (control) or three different whole oilseeds added (% of inclusion in the diet): soybean (24.22%), sunflower (26.88%), and cottonseed (25.23%), as described in the Table 1. In the soybean grain diet, soybean meal was replaced by soybean oil to achieve the 7% ether extract (EE), as recommended for grain diets by National Research Council (21). The diets were formulated according to NRC (21). For diets with grains, 150 g/kg of crude protein (CP) and 70 g/kg of ether extract (EE) in dry matter (DM) basis were determined (Table 1).

**Table 1:** Ingredients and chemical composition of experimental diets.

	**Diets**			
	**Control**	**Cottonseed**	**Soybean**	**Sunflower**
**Ingredients**	**g/kg DM**			
Corn silage	400.0	400.0	400.0	400.0
Corn	410.8	259.1	339.2	180.7
Soybean meal	174.2	73.5	0.0	135.5
Oilseed grains	–	252.3	242.2	268.8
Soybean oil	–	–	3.6	–
Mineral premix*	15.0	15.0	15.0	15.0
**Chemical composition (g/kg DM)**				
Dry matter (g/kg)	508.9	511.1	511.0	510.7
Organic matter	946.9	944.8	946.9	939.6
Crude Protein	150.0	150.0	150.0	150.0
Neutral detergent fiber	302.8	381.5	314.1	345.3
Ether extract	24.7	70.0	70.0	70.0
Fatty Acids**(g/kg DM)				
C14:0	0.2	0.6	0.3	0.1
C16:0	3.6	13.5	8.5	4.9
C16:1*n*−7	0.2	0.5	0.3	0.2
C18:0	1.3	3.4	3.6	3.2
C18:1*n*−9	6.5	13.2	15.0	16.7
C18:2*n*−6	9.1	32.6	31.5	39.4
C18:3*n*−3	0.1	0.9	3.5	0.6
Others	2.7	2.2	4.1	1.8
∑ Saturated	5.1	17.5	12.5	8.2
∑ Unsaturated	15.9	47.3	50.3	56.9

*DM, Dry matter*.

*Composition: sodium 100 g/kg; phosphor 88 g/kg; calcium 176 g/kg; magnesium 8,000 mg/kg; sulfur 22 g/kg; zinc 3,000 mg/kg; copper 1,000 mg/kg; cobalt 80 mg/kg; iodine 60 mg/kg; selenium 20 mg/kg; fluorine 880 mg/kg.

**Values calculated from compilation of literature data (16–19) and Brazilian Cattle Feed Composition Table (20).

The animals were slaughtered with an average body weight of 518 ± 22.93 kg, in a commercial abattoir following the Brazilian legislation (22). After slaughter, the carcasses were cooled to 0–2°C for 24 h and then deboned. Samples of longissimus thoracis (LT), trimmed of subcutaneous and intermuscular fat, were collected from each left–half carcass, between the ninth and sixth ribs. Six samples of the LT were collected from each experimental diet, totalizing 24 samples, vacuum packed in polyethylene plastic bags and kept frozen (−20 ± 2°C) until burgers processing.

### Burger processing

Burgers were prepared according to the Burger Technical Identity and Quality Regulations (23), that regulate the industrialization of animal products and the standardization of processing. The limits for the physicochemical characteristics were as follows: maximum 23% fat, minimum 15% protein, 3% total carbohydrates, 0.1% calcium in raw burgers, and 0.45% calcium in cooked burgers. The sensory characteristics of color, flavor, aroma, and texture were defined according to the product processing (23).

The burgers corresponding to each diet (control, soybean, sunflower, and cottonseed) were prepared from six samples of LT from each treatment. The pooled sample was ground in an industrial meat grinder (CAF, model 22, Rio Claro, São Paulo) fitted with a 5 mm diameter disc. After grinding, the samples were homogenized again, and a 10 kg aliquot of ground meat was removed; ice and sodium polyphosphate (10 and 0.5% of the weight, respectively) were subsequently added.

The mixture was kept in a cold refrigerated chamber at 0–2°C during the molding process. Each burger was made using a 100 g of the mixture molded into plastic shapes (10 cm diameter). After molding, the burgers were individually wrapped in 0.006 μm polyethylene plastic bags (15 × 30 cm) and sealed with electrical tape. Each treatment was kept in separate aluminum trays and stored at −18°C for 0, 30, 60, 90, and 120 days.

**Figure 1:**
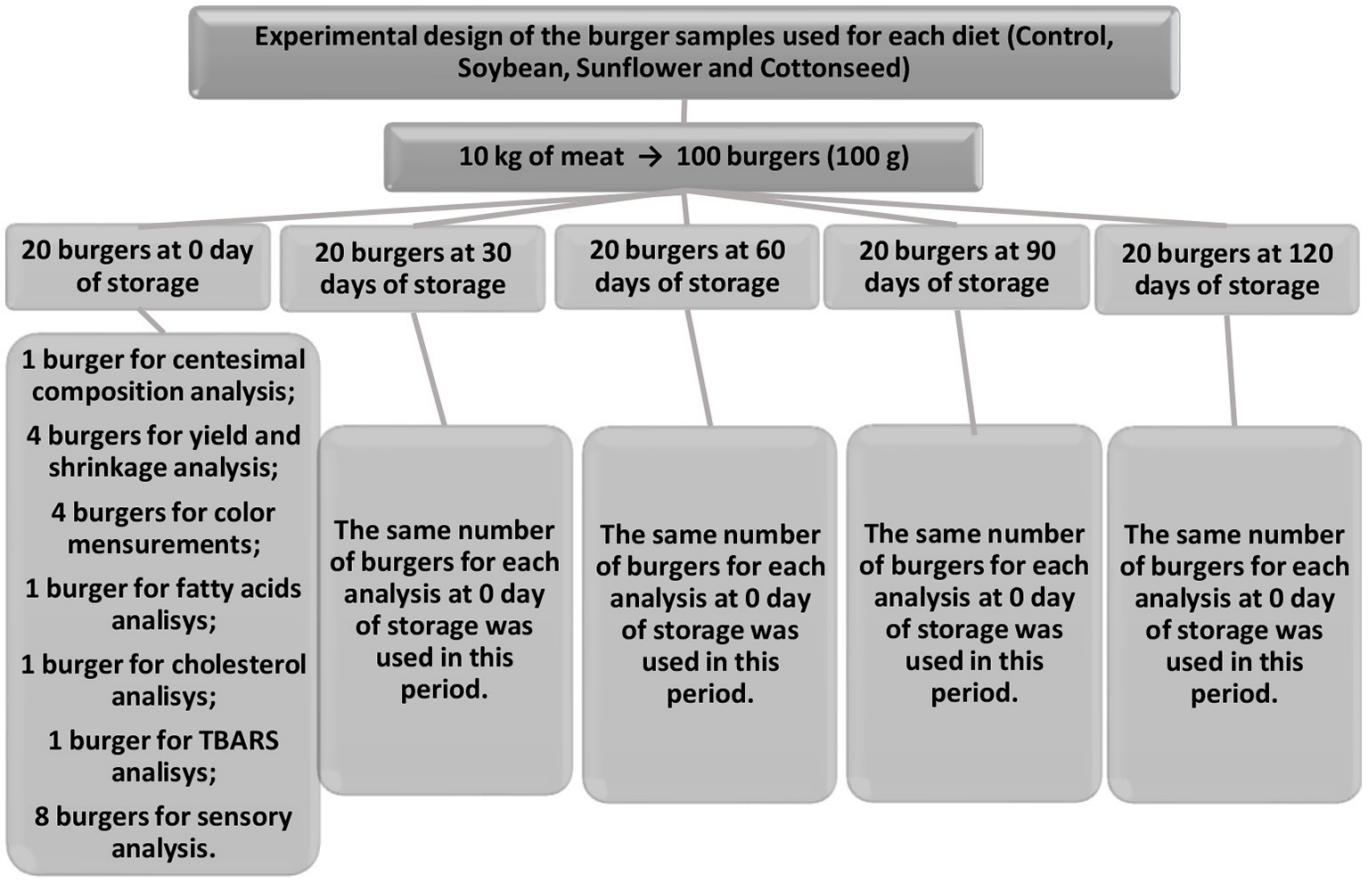
Quantity and distribution of burgers in the diets and centesimal, instrumental and sensory analyses.

A total of 100 burgers were prepared for each dietary treatment, with 20 burgers allocated to each storage period (0, 30, 60, 90, and 120 days). Then, eight were used for the sensory analysis, one for centesimal composition, one for fatty acid profile, one for cholesterol analysis, one for the determination of compounds reactive to 2-thiobarbituric acid (TBARs), four for cooking yield and shrinkage percentage, and four for color and pH evaluation, totalizing 20 burgers analyzed for all these traits for each diet and storage period (Figure 1).

### Laboratorial analysis

#### Centesimal composition

Analyses of protein, moisture, and fixed mineral residue content were carried out according to AOAC (24) 976.05, 930.15, and 942.05, respectively. The EE analysis was performed according to the Am 5–04 methodology of AOCS (25), in an automatic extraction system (Ankom XT14 Extractor, ANKOM^®^ Technology, Macedon, NY).

#### pH

The pH of the burgers was determined using a portable pH probe (HI 99163, Hanna^®^ instruments, São Paulo, Brazil), with a pre–amplified penetration electrode (FC232D). Measurements were made in the center of the burgers.

#### Color

To determine the color parameters the burgers were removed from the packaging and exposed to oxygenation for 20 min. The color measurements were performed with a colorimeter (Chroma Meter CR400, Konica Minolta^®^, Osaka, Japan), with D65 illuminant and 10° as the standard observation points. We used the CIELAB color space, in that L^*^ corresponds to the lightness content, a^*^ to the redness and b^*^ to the yellowness. The final L^*^a^*^b^*^ values were obtained by the average of three scans in distinct points of the sample.

#### Cooking yield and shrinkage percentage

Analysis of cooking yield and shrinkage percentage were performed according to Berry (26). Before cooking, the burgers were weighed, and the diameter measured using an Eccofer metal mechanical caliper. The burgers were grilled on an iron plate placed directly on a flame: four samples of the same dietary treatment were placed side by side on the plate and the temperature was measured with a skewer–type thermometer (Incoterm, model 9791, Porto Alegre, Rio Grande do Sul), positioned in the geometric center of the burgers. When the temperature reached 42°C, the burgers were turned, and then removed from the plate when they reached 71°C. The plate was washed and sanitized between cooking burgers from each dietary treatment. After cooking, the samples were weighed and measured again to calculate the yield and shrinkage percentage after cooking, respectively.

#### Fatty acid profile, lipid index, and cholesterol

Lipid extraction of burger samples was performed following the methods of Folch et al. (27), using chloroform/methanol, and a internal standard of methyl ester of n–nonadecanoic acid (C19:0) for fatty acid normalization. The lipid extracts were converted to fatty acid methyl esters (FAME), according to Nürnberg et al. (28). Samples were analyzed using a gas chromatography (Thermo Trace GC Ultra chromatograph) equipped with flame ionization detector and a column of stationary phase (SP-2560, Merck/Sigma-Aldrich, Supelco^®^, Bellefonte, PA, USA), heated under the following conditions: initial temperature 100°C, hold for 5 min, ramp of 4°C/min, final temperature of 220°C, hold for 30 min; post–race: maximum ramp up to 100°C, hold for 5 min. A split mode injector was used with the following conditions: a 1:10 ratio, temperature held at 260°C, flow set to 13 mL, and helium used as a carrier gas. The FAMEs were identified by a comparison of the FAME retention times with those of authentic standards (FAME mix components, Supelco^®^, Bellefont, PA, USA) following the same injection method. The mobile phase used nitrogen at a constant flow of 1.3 mL/min, and a sample volume of 3.0 μL. ChromQuest 5.0 software (Thermo Fisher Scientific) was used for analysis of the chromatograms.

Thrombogenicity and atherogenicity indexes were calculated according to Ulbricht and Southgate (29). Stearoyl CoA Desaturase (SCD) activity at C16 (SCD−16), C17 (SCD−17) and C18 (SCD−18) was calculated according to Guerreiro et al. (30). Elongase activity was determined according to Malau–Aduli et al. (31). The hypercholesterolemic:Hypocholesterolemic (h:H) relationship was determined according to the methods of Santos–Silva et al. (32), and the desirable fatty acids and *n*−6/*n*−3 ratio quantified according to Rhee (33). Cholesterol was measured according to the methods of Saldanha et al. (34).

#### Determination of TBARs

To determine compounds reactive to TBARs, the samples were analyzed by the aqueous acid extraction method adapted by Vyncke (35). TBARs were measured as mg of malonaldehyde per gram of sample.

#### Sensory analysis

The ethics committee on research in human beings (CEP/UFMS, protocol N° 2.746.173) approved this project for consumer analysis.

The sensory analysis was undertaken according to the American Meat Science Association (36) for the evaluation of the sensory panel for consumers. There were five panels, one for each storage period (day 0, 30, 60, 90, and 120 days). Each panel was composed of at least 109 people, totalizing (545 judges). Men and women, aged between 18 and 50 years were recruited from the Faculty of Veterinary Medicine and Animal Science in the Federal University of Mato Grosso do Sul. Once they had accepted the invitation, received the informed consent form containing information on the burger composition and the voluntary participation process. The forms were signed for research validation. Groups of 12 evaluators were accommodated in individual booths, with a table and chair in an air–conditioned room at 23°C. The booths were arranged so there was no visual contact between the judges. In each booth, a glass of water at room temperature (23°C) and a salt biscuit was available for cleaning the palette when necessary, and a paper napkin and plastic cup for disposal of the samples after the test. The panelists were orientated to discard de samples after shewing, if they want, not being necessary the deglutition after the evaluation.

The preparation of the samples for the test consisted of grilling the burgers on an iron plate (eight burgers from each diet) placed directly over a flame. The temperature was monitored by a skewer–type thermometer (Incoterm, model 9791). The burgers were turned when they reached 42°C and removed from the plate when they reached 71°C. Immediately after cooking, each burger was divided into equal sub samples, approximately nine grams each. Two sub samples (one sample splited in two parts) of the burger of each diet were placed in glass cups, identified with the sample codes that had been cleaned and sanitized with products that did not affect the taste or odor, and covered with aluminum foil to maintain the aroma and remained heated at 49°C until the evaluation.

The samples were offered to the tasters in a random, monadic, coded order for the evaluation of flavor, aroma, and juiciness attributes, with scores ranging from 1 to 7 points, with 1 meaning “very bad”, 4 corresponding to “neither good / nor bad” and 7 being “excellent” (36). The judges were asked which sample they liked the most and the least as a representation of global acceptance.

### Statistical analysis

The pH, color, yield, shrinkage, and TBARs, data were analyzed with a generalized linear model (GLM) in SAS OnDemand for Academics, by an analysis of variance (ANOVA) using the scheme factorial 4 diets (Control, Soybean, Sunflower, and Cottonseed) × 5 storage periods (0, 30, 60, 90, and 120 days). Fatty acids were analyzed only for diets effects. When significant, data were then analyzed by a Tukey's test to compare means. For the evaluation of dietary oilseeds on consumers sensory panel, the variables were analyzed by Wilcoxon test, considering Monte Carlo Estimate for the Excat Test with 95% Confidence Limits. When significant, Wilcoxon scores were subjected Pairwise Two-Sided Multiple Comparison by DSCF (Dwass, Steel, Critchlow-Fligner) method. Values of *p* < 0.05 were considered significant. The judges' responses regarding global acceptance of diet factor were represented by a heat map (SAS OnDemand for Academics).

## Results

### Centesimal composition

Diet had a significant effect on the centesimal composition of burgers (*p* < 0.05) (Table 2). The meat of the animals fed with soybean resulted in burgers with a higher percentage of crude protein (21.80%) when compared to those fed with sunflower (20.52%) (*p* < 0.05), and presented higher percentage of ether extract (4.43%) when compared to animals fed with the control diet (3.13%), sunflower (3.09%) and cottonseed (3.29%). On the other hand, the lowest moisture content was observed in burgers produced with meat from the sunflower diet (75.08%) (*p* < 0.05). The ash content was different (*p* < 0.05) only among the burgers produced with soybean (1.31%), control (1.13%) and sunflower (1.19%), in which the highest value was observed in the burgers produced with the meat from the soybean diet. The ash content of the burgers produced from the meat of animals fed with cottonseed was the same as those fed with the other diets (*p* > 0.05).

**Table 2:** Centesimal composition of burgers of beef cattle fed with different sources of oilseed grains.

**Item**	**Diets**					
**(%)**	**Control**	**Soybean**	**Sunflower**	**Cottonseed**	**SEM**	***p*–value**
Protein	21.52^ab^	21.80^a^	20.52^b^	21.47^ab^	0.289	<0.000
Moisture	76.15^a^	75.08^b^	76.48^a^	76.11^a^	0.236	<0.000
Ash	1.13^b^	1.31^a^	1.19^b^	1.22^ab^	0.026	<0.000
Ether extract	3.13^b^	4.43^a^	3.09^b^	3.29^b^	0.539	0.046

*SEM, standard error of the mean*.

^a, b^*Means in the same row with different letters differ according to Tukey test (p < 0.05)*.

### Color, pH, yield, and shrinkage rate

There was an effect of the diet and of the storage period on the pH, without interaction between main effects (*p* < 0.05) (Table 3). The pH was higher in burgers produced with oilseeds: soybean (6.03), sunflower (6.02) and cottonseed (5.98), and in storage times 60 (6.12) and 120 days (6.05). However, pH at 120 days of storage did not differ (*p* > 0.05) among 0 (5.93) and 30 days (5.93).

**Table 3:** Instrumental characteristics of beef burgers subjected to different diets and storage period.

**Item**	**Diets**				**Storage time (days)**					***p*** **-value**			
	**Control**	**Soybean**	**Sunflower**	**Cottonseed**	**0**	**30**	**60**	**90**	**120**	**Diet**	**Period**	**Interaction**	**SEM**
pH	5.88^b^	6.03^a^	6.02^a^	5.98^ab^	5.93^bc^	5.93^bc^	6.12^a^	5.84^c^	6.05^ab^	0.005	<0.000	0.512	0.125
L	33.71	34.09	34.58	34.99	34.99	35.57	33.88	34.60	32.68	0.686	0.195	0.116	3.553
b*	10.97	12.10	11.93	10.94	9.74^b^	12.43^a^	11.63^ab^	11.74^ab^	11.90^ab^	0.256	0.024	0.412	2.347
Y%	70.10^b^	70.70^ab^	71.20^ab^	71.70^a^	69.25^c^	71.25^ab^	71.00^ab^	72.37^a^	70.75^bc^	0.016	0.000	0.959	1.037
S%	15.20	13.95	13.75	13.45	15.06^a^	13.12^ab^	14.93^ab^	12.68^b^	14.62^ab^	0.128	0.018	0.576	2.450

*L, Luminosity; Y, Yield; S, Shrinkage*.

*SEM, standard error of the mean*.

^a, b, c^*Means in the same row with different letters differ according to Tukey test (p < 0.05)*.

The storage period influenced the color parameters of the burgers (*p* < 0.05) (Table 3). The b^*^ value was higher at 30 days of storage (12.43) compared 0 day of storage (9.74). There was an interaction between different diets and storage period in beef burgers on the a^*^ parameter (Table 4). Burgers had a higher a^*^ value at 30 days of storage (21.98) compared to 0, 60 and 90 days (*p* < 0.05). The diets influenced the a^*^ parameter only when the burgers were subject at 30 and 120 days of storage.

**Table 4:** Effect of storage period on intensity of the a^*^ parameter in burgers produced by meat of steers fed with different oilseeds sources, and interactions between factors.

**Period (days)**					**Means of storage period**
	**Control**	**Soybean**	**Sunflower**	**Cottonseed**	
0	15.81^C^	13.84^C^	16.77^B^	15.47^B^	15.47^C^
30	22.55^Aab^	23.96^Aa^	21.16^Aab^	20.25^Ab^	21.98^A^
60	19.83^AB^	19.40^B^	19.56^AB^	18.48^AB^	19.31^B^
90	17.88^BC^	16.54^BC^	17.98^AB^	18.81^AB^	17.79^BC^
120	18.05B^Cbc^	23.61^Aa^	21.09^Aab^	16.20^Bc^	19.73^AB^
Means of diet	18.82	19.46	19.31	17.83	–

*p-values: Diet 0.202; Storage period < 0.01; Interaction (diet × storage period) = 0.043*.

Standard error of the mean for a* parameter = 2.608.

^a, b, c^*Means in the same row with different letters differ according to Tukey test (p < 0.05) and express the effect of the diet*.

^A, B, C^*Means in same row with different capital letters differ by Tukey test (p < 0.05) and express the effect of the period of storage*.

The inclusion of oilseeds in the animal diets also influenced the yield of the burger (*p* < 0.05). The cottonseed–containing diet showed a higher average yield when compared to burgers produced from animals that did not consume oilseeds (Table 3). Yield and shrinkage rates were also influenced by the storage period (*p* < 0.05). Burgers kept for zero days on frozen storage presented lower yield compared to those kept 30–90 days on frozen and higher shrinkage percentage compared to burgers stored for 90 days.

### Fatty acid profile

There was no difference (*p* > 0.05) in the proportion of saturated (C15:0, C16:0, C17:0, C18:0, C21:0, C22:0 and C24:0), monounsaturated (C16:1, C17:1c−9, C18:1t−11, C18:1c−9, C18:1t−10), and polyunsaturated (C18:2*n*−6c, C18:3*n*−3, C20:3*n*−6, C20:5, C22:2*n*−6, C22:6*n*−3) fatty acids in burgers from animals fed different diets (Table 5).

**Table 5:** Fatty acid composition and cholesterol content (mg 100 g^−1^ of muscle) of burger of the steers fed different oilseeds grains.

**Fatty acids (FA)**	**Diets**				**SEM**	***p*** **–value**
	**Control**	**Soybean**	**Sunflower**	**Cottonseed**		
C14:0	2.89^ab^	6.82^a^	3.64^ab^	0.66^b^	0.81	0.051
C15:0	1.44	0.72	1.38	1.66	0.19	0.330
C16:0	137.96	122.77	128.16	76.13	13.28	0.347
C16:1	11.91	12.68	8.39	3.86	1.47	0.101
C17:0	6.96	5.57	6.83	5.13	0.69	0.749
C17:1c−9	2.88	2.50	2.20	1.01	0.30	0.193
C18:0	130.44	82.08	114.24	76.82	11.90	0.309
C18:1t−11	6.04	5.62	4.78	5.74	0.72	0.936
C18:1c−9	104.62	95.94	106.65	46.59	11.09	0.161
C18:1t−10	11.20	7.54	6.71	6.60	0.87	0.166
C18:2*n*−6c	55.04	41.83	43.86	31.44	0.87	0.207
C18:3*n*−3	3.77	2.92	2.92	1.96	0.28	0.118
C20:3*n*−6	4.21	3.36	5.90	2.71	0.49	0.118
C20:5	3.44	2.11	4.09	–	0.38	0.109
C21:0	2.50	2.46	2.48	0.78	0.34	0.583
C22:0	2.07	1.68	2.17	1.39	0.17	0.433
C22:2*n*−6	1.01	0.44	1.22	0.89	0.14	0.122
C22:6*n*−3	1.05	0.62	1.15	1.37	0.12	0.302
C24:0	12.52	8.07	13.27	9.56	1.12	0.339
∑ SFA	297.47	202.81	276.95	170.09	25.21	0.221
∑ MUFA	135.79	129.24	139.69	62.54	14.58	0.177
∑ PUFA	64.65	49.47	52.94	35.38	4.94	0.157
*n*−3	4.67^a^	3.44^ab^	3.96^ab^	2.19^b^	0.34	0.038
*n*−6	59.99	46.03	48.98	33.50	4.63	0.186
*n*−6/*n*−3	12.69	13.55	11.85	15.37	0.644	0.294
TI	2.33	2.13	1.94	2.73	0.12	0.096
IA	0.72	0.80	0.61	0.74	0.03	0.104
Desirable FA	330.88	233.43	337.39	174.75	29.74	0.139
h:H	1.20^ab^	1.12^ab^	1.43^a^	1.08^b^	0.05	0.043
SCD−16	7.86^a^	8.86^a^	7.72^a^	4.80^b^	0.48	0.007
SCD−17	72.36	67.82	77.49	78.90	1.60	0.058
SCD−18	54.08	55.49	53.12	61.05	1.49	0.192
Elongase	62.26	63.13	64.14	62.78	0.86	0.895
Cholesterol	55.95	55.70	56.02	62.64	3.62	0.124

TI (thrombogenicity index): [(ΣC14:0, C16:0, C18:0)/Σ(0.5 × ΣMUFA), (0.5 × Σn−6), (3 × Σn−3), (Σn−3/Σn−6)]; AI (atherogenicity index): {[(4xC14:0), C16:0]/[(Σn−3, n−6) + (C18:1c−9) + (Σ others MUFA)]}; h:H (hypocholesterolemic:hypercholesterolemic index): (ΣC18:1c−9, C18:2c−9, C18:3n−6, C20:5 / ΣC14:0, C16:0); SCD−16 (stearoyl CoA desaturase in C16:0): (c9–16:1/(c9–16:1 + 16:0)^*^100); SCD−17 (stearoyl CoA desaturase in C17:0): (c9–17:1/(c9–17:1 + 17:0)^*^100); SCD−18 (stearoyl CoA desaturase in C18:0): (c9–18:1/(c9–18:1 + 18:0)^*^100).

^a, b^*Means in the same row with different letters differ according to Tukey test (p < 0.05)*.

The myristic acid (C14:0) concentration was influenced by the inclusion of oilseeds (*p* < 0.05). burgers made by the meat of animals fed with soybean showed higher concentrations than those fed cottonseed.

The concentration of *n*−3 was different in burgers produced with meat from cattle fed without oilseeds and cottonseed (*p* < 0.05). However, the *n*−6 concentration and the *n*−6/*n*−3 ratio in the burgers were not influenced by the presence of oilseeds in the bovines diets. The thrombogenicity, atherogenicity indexes and cholesterol content did not differ between treatments (*p* < 0.05).

The h/H ratio varied significantly between burgers made from meat of the animals fed different diets (*p* < 0.05) were the burgers from animals fed control, soybean and sunflower diets had highest h/H values when compared to those cottonseed. SCD−16 activity decreased in the tissue of animals fed with cottonseed, compared to other diets (*p* < 0.05).

### Lipid stability

The lipid stability of burgers was influenced by diets with grains rich in unsaturated fatty acids (*p* < 0.05) and gradually increased with the storage time (*p* < 0.05) (Table 6). The sunflower formulation diet provided burgers with smaller lipid oxidation (Table 6).

**Table 6:** Effect of storage period on 2–thiobarbituric acid reactive substance (TBARS, mg/kg of malondialdehyde) in burgers produced by meat of steers fed with different oilseeds sources, and interactions between factors.

**Period**	**TBARS**				**Means of**
**(days)**	**(mg/kg of**				**storage**
	**malondialdehyde)**				**period**
	**Control**	**Soybean**	**Sunflower**	**Cottonseed**	
0	0.46^E^	0.40^E^	0.40^E^	0.47^E^	0.43^E^
30	0.75^Da^	0.57^Bb^	0.53^Db^	0.74^Da^	0.64^D^
60	1.18^Ca^	1.23^Ca^	1.04^Cb^	0.97^Cb^	1.10^C^
90	1.40^Ba^	1.41^Ba^	1.24^Bb^	1.42^Ba^	1.36^B^
120	2.03^Aa^	2.00^Aa^	1.96^Aab^	1.90^Ab^	1.97^A^
Means of diet	1.17^a^	1.12^ab^	1.03^c^	1.10^b^	-

*p-values: Diet < 0.0001; Storage period < 0.001; Interaction (diet × storage period) = 0.002*.

*Standard error of the mean for TBARS = 0.045*.

^a, b, c^*Means in the same row with different letters differ according to Tukey test (p < 0.05) and express the effect of the diet*.

^A, B, C, D, E^*Means in same row with different capital letters differ by Tukey test (p < 0.05) and express the effect of the period of storage*.

There was an interaction (*p* < 0.05) between diets and storage period on lipid stability of burgers. The TBARS value of the burgers before the storage (day 0) were similar (*p* > 0.05). At 30 days of storage, TBARS values were similar among burgers of bovines that consumed soybean and sunflower, which showed the lowest lipid oxidations (0.57 and 0.53, respectively) when compared to the control and cottonseed diets (0.75 and 0.74, respectively).

When the burgers were submitted to 60 days of storage, the diets without oilseeds and with soybean provided higher TBARS (*p* < 0.05) in the burgers, while sunflower and cotton reduced lipid oxidation. In burgers stored for 90 days sunflower presented lower value of TBARs.

All the diets provided burgers with more lipid oxidation at 120 days of storage. The control, soybean and sunflower diets presented the highest values of lipid oxidation (*p* < 0.05) in this period.

### Sensory analysis

There was a significant effect of dietary oilseeds on the sensory aspects of burgers (*p* < 0.05): burgers from cottonseed–fed cattle were less acceptable for flavor, and odor (*p* < 0.05). Whereas, burgers produced with sunflower seed was rated the most succulent (*p* < 0.05) (Table 7).

**Table 7:** Sensory characteristics of grilled burgers from steers fed diets with oilseeds and different storage times (0, 30, 60, 90, and 120 days).

**Item**	**Diets**				**Storage period (days)**					***p*** **-value**		
	**Control**	**Soybean**	**Sunflower**	**Cottonseed**	**0**	**30**	**60**	**90**	**120**	**Diet**	**Period**	**SEM**
Flavor	4.94^a^	5.08^a^	5.04^a^	4.35^b^	5.05^a^	4.95^ab^	4.81^abc^	4.78^bc^	4.70^c^	<0.000	<0.000	0.026
Aroma	4.94^b^	5.06^ab^	5.13^a^	4.67^c^	5.10^a^	4.95^ab^	4.79^b^	4.94^ab^	4.96^ab^	<0.000	0.003	0.023
Juiciness	4.85^b^	4.83^b^	5.03^a^	4.80^b^	5.14^a^	5.10^ab^	4.83^b^	4.77^bc^	4.61^c^	0.007	<0.000	0.027

*SEM, standard error of the mean*.

*Sensorial ratings for flavor, aroma, and juiciness: 1 to 7 points, with 1 meaning “very bad”, 4 corresponding to “neither good / nor bad” and 7 being “excellent”*.

^a, b, c^*Means in the same row with different letters differ according to Dwass, Steel, Critchlow-Fligner test (p < 0.05)*.

The consumer analysis showed a variation in the acceptable storage period for grilled burgers (*p* < 0.05): acceptance of burgers decreased over storage time. Storage between 60 and 120 days scored lower averages in sensory acceptance for the attributes for overall quality (*p* < 0.05) (Table 7).

**Figure 2:**
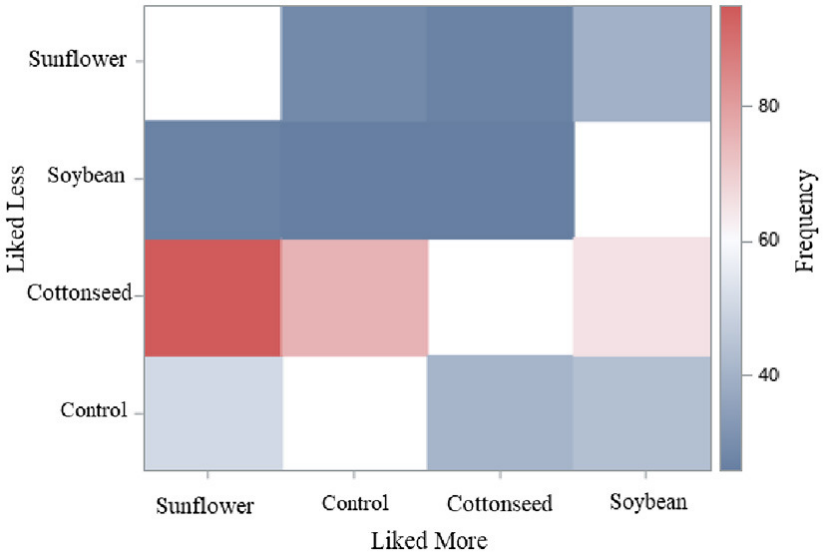
Heat map with the frequency of responses from judges for global acceptance on sensory analysis of burgers. In the blind test, judges answered which sample liked more and liked less between burgers of steers without or with different oilseed grains sources in diet: control, soybean, cottonseed, and sunflower. The frequency of combined responses among liked more (*x*) and liked less (*y*) is represented by the intensity of colors blue (cold) to red (hot). The absence of color (white) represents the comparison of a diet with itself.

The heat map demonstrated a combination of judges' responses to “like more” and “like less” of burgers made with meat from animals fed with or without sources of oilseeds. The map showed that panelists more frequently responded that “liked more” and “liked less” the burgers from steers fed sunflower or cottonseed, respectively (Figure 2). These results indicate high acceptance for burgers containing meat from cattle fed diets containing sunflower or soybean, and low acceptance for those containing cottonseed.

## Discussion

### Centesimal composition

The inclusion of soybean oil (0.36%) in the soybean diet may have contributed to the observed increase in crude protein and EE, providing the selection of certain microorganisms in the rumen environment, thereby inhibiting gram–positive bacteria and favoring a greater deposition of muscle tissue. Soybean degrades very quickly, which increases its availability to digestive enzymes and ruminal bacteria and leads to a higher amino acid concentration (37).

The higher EE content in the burgers from animals fed the soybean diet may be related to the addition of soybean oil. The use of oilseed grains in their integral form acts as a natural barrier that protects the lipid content of the seed and allows the fat to be released slowly in the rumen. On the other hand, the supply of oil increases the capacity of microorganisms to saturate the PUFAs of the grains (38). Selectivity occurs in the absorption process, and UFAs are esterified with cholesterol esters and phospholipids, and they are not subjected to hydrolysis by lipoprotein lipase. These UFAs are located mainly on cell membranes. When animals need energy, fatty acids from adipose tissue are mobilized to meet the energy requirements; therefore, fatty acids found in meat are beneficial to human health (39).

### Color, pH, yield, and shrinkage rate

The high pH (6.0) observed in our study may be associated with the sodium polyphosphate in the burger formulation, since this additive has the function of increasing the water holding capacity (WHC), yield, and the juiciness of meat products. The increase in the WHC changes the isoelectric point of the proteins, raising the pH of the food.

A reduction in yield and a higher degree of shrinkage is directly associated with the water holding capacity, because when meat is cooked, the muscle proteins denature, resulting in the shrinkage of the fibers and the loss of water (40).

The effects observed on the a^*^ parameter during the different periods of storage may be due to the oxygen availability in the package (41). Exposing meat to oxygen lead to transformation of deoxyhemoglobin in oxymyoglobin, giving to the muscle tissue a bright red color. In the absence of oxygen, oxymyoglobin deoxygenation occurs, changing the state of the pigment to reduced myoglobin: this increases susceptibility to oxidation and produces the brown–colored pigment, called metmyoglobin (42). Metmyoglobin formation increases in meat stored for longer storage periods, and this pigment tends to reduce the intensity of the red coordinate (a^*^) of meat (43). This phenome could be the responsible for the reduction in the red intensity, observed in this study, in the burgers stored for more than 30 days. The interaction between the diets and time on storage in a^*^ coordinate obtained in our study is unclear, since hemoglobin tends to lose intensity during prolonged storage period (44). One hypothesis could be attributed to the different antioxidant capacity of the diets, indicated by the different values of TBARS along the storage periods, that could be responsible for reducing the generation of reactive oxygen species (ROS) in the burgers, impacting directly on the amino acids and proteins oxidation and, consequently, on the myoglobin reduction capacity. The increase in the b^*^ value could be also associated to the oxidation of this pigment, as the when it oxidizes, myoglobin becomes brown–colored metmyoglobin, and the b^*^ value increases. An increase in this coordinate is associated with the increase in yellow-brown pigments (45).

### Fatty acid profile

Despite increasing the supply of PUFA (Table 1), burgers showed higher concentrations of SFA. This is related to ruminal biohydrogenation carried out by microorganisms, even when the animals are supplied with lipids in the form of grains or by lipid oxidation during the processing of the burgers (46, 47).

The C14:0 and C16:0 increase blood cholesterol of consumers, with C14:0 having an effect four times greater than C16:0 (3). However, in this study, the proportion of myristic acid (C14:0) in meat was, on average, 20 times lower that than of palmitic acid (C16:0) (Table 4). This is beneficial to human health, given the lower cholesterolemic potential of C16:0. Stearic acid (C18:0) was identified in all evaluated diets, at an average concentration of 115 mg/100 g. This fatty acid is characterized by not having hypercholesterolemic properties, therefore it is positive in terms of human and cardiologic health (2).

The presence of oleic acid (C18:1 *cis* 9) in beef is due to the incomplete biohydrogenation of dietary UFAs, as well as the endogenous desaturation of stearic acid (C18:0) by SCD−18 (48). The increase in the concentration of oleic acid is highly desirable due to its hypocholesterolemic effect. Foods rich in oleic acid help to reduce the levels of total cholesterol and the percentage of LDL, and improve the ratio of LDL and HDL in humans (49). In this study, diets with oilseeds did not result in higher concentrations of oleic acid.

SCD activity is determined by the relationship between the product and the substrate, therefore, greater amounts of palmitoleic acid in meat are associated with greater SCD activity (50). This promotes the desaturation of C16:0 in C16:1 acid, and consequently increases the proportion of monounsaturated fatty acids (51). In this study, the highest concentration of C16:0 was from the diet containing cottonseed (13.5 g/kg DM, Table 1), and the C16:0 and C16:1 acid concentration was similar in burgers.

The h/H ratio assesses the type and amount of fatty acids present in meat, and their influence on cholesterol transport in the body. The UFAs oleic, linoleic, arachidonic, linolenic, eicosapentaenoic acid (EPA), docosapentaenoic acid (DPA), and docosahexaenoic acid (DHA) are considered hypocholesterolemic, as they help prevent cardiovascular diseases by reducing the amount of LDL in the bloodstream (32). Our results suggest that burgers produced from animals fed sunflower seeds are healthier than those produced from cottonseed–fed animals.

Fatty acids of the *n*−6 and *n*−3 family are essential and compete for enzymes associated with the process of desaturation and chain elongation (52). Although enzymes have a greater affinity for the *n*−3 family, the conversion of alpha–linolenic (AL) acid to PUFA is affected by the amount of dietary linoleic acid (LA) (53). The higher concentration of LA in the oilseed diet could have provided higher concentrations of AL and consequently increased the *n*−3 content in the burgers. However, there was no effect of diet on the LA concentration, whereas the control, soybean, and sunflower treatments produced burgers with higher amounts of *n*−3 fatty acids. According to Ponnampalam et al. (54), the inclusion of *n*−3 in the diet of ruminants does not guarantee increased fatty acids in the meat products, due to the ruminal biohydrogenation process.

Although variation in the concentration of *n*−3 fatty acids was observed, there was no difference in the *n*−6/*n*−3 ratio in burgers from animals fed oilseed grains (*p* > 0.05). The *n*−6/*n*−3 ratio is important for human health (55), although there are no specific recommendations for *n*−6/*n*−3 values, the Food and Agriculture Organization of the United Nations (56) recommends to consume a balanced dietary intake by. The recommended intake of *n*−3 varies according to age, dietary standard, and physiological status (57). The *n*−6/*n*−3 values in this study were higher than the values of 4:1 to 10:1 and 5:1 suggested by the Canadian (58) and Nordic (59) health committees, respectively.

The thrombogenicity index rates the saturated fatty acids C14:0, C16:0, and C18:0 as being responsible for the thrombogenic potential, and the levels of UFAs and PUFAs as antithrombogenic. Likewise, the atherogenicity index assesses the proportion of acids (lauric, palmitic, and myristic) in relation to the sum of monounsaturated fatty acids and the sum of fatty acids from the omega 3 and 6 family that have anti–atherogenic properties. These indicators quantify the potential for stimulating platelet aggregation, as lower the values of the thrombogenicity and atherogenicity indexes, lower the risk of developing cardiovascular disease is (60). In this study, the results of these indices suggest that the inclusion of oilseeds does not affect these characteristics, and all the evaluated burgers are equally beneficial to human health.

Cholesterol content is related with the level of saturated fatty acids. In this study, the cholesterol concentrations were the same as the saturated fatty acids concentration. Santos Filho (3) determined that a daily intake of 300 mg cholesterol was adequate for optimal human health. Therefore, the cholesterol content of the burgers in this study, produced from all diets, are suitable for consumption. Considering the average cholesterol level of 57 mg/100 g of burger found in this study, it would be possible to consume 526 g of burgers to reach the recommendation of Santos Filho (3).

### Lipid stability

Differences in meat lipid stability may be associated with *n*−3 content (61). The *n*−3 conformation has a greater number of double bonds than those of *n*−6 and *n*−9 and, therefore, could be more susceptible to lipid peroxidation (53). Oxidation is a very complex chemical reaction, which depends on catalytic action (temperature, pH, metal ions, free radicals) and is divided into three phases: initiation, propagation, and termination. At initiation, free radicals are released from fatty acids by separating the hydrogen atom that is located between the double bonds. In the propagation, the elimination of a hydrogen atom, or the addition of oxygen to an alkyl radical occurs, forming primary products, such as peroxides and hydroperoxides. In the terminal phase of the reaction, free radicals combine to form by–products, such as aldehydes, alcohols, and other volatile and non–volatile compounds (62). In our study lipid stability was lower in burgers produced with beef from sunflower-fed bovine. Likewise had a lower concentration of *n*−3 compared to burgers from animals that did not intake oilseeds.

Regarding to oxidation gradually increased with storage time in this study, malonaldehyde levels in burgers were highest at 120 days of storage, close to 2 mg/kg. This level of oxidation causes a rancid flavor and odor in meat, which can be identified by sensory analysis (63). Therefore, even when stored at negative temperatures, enzymatic reactions still occur in the lipid fraction of meat and can lead to the process of oxidative rancidity and development of the off-flavors in the meat and meat products (10).

Oxidation is one of the storage problems related to the oxidation of color of the meat. This because the exposure of meat to oxygen causes the pigment reaction and produce the oxymyoglobin, which gives a bright red color to muscle tissue. In the absence of oxygen, oxymyoglobin deoxygenation occurs, changing the pigment to reduced myoglobin. This change in state increases susceptibility to oxidation and produces the brown–colored metmyoglobin (42). The oxidative reaction occurs even when the meat is stored at negative temperatures (−9, −13, and −18°C) (64).

Oxidative reactions in meat products mainly occur in the deboning, processing, and storage stages because processing breaks down the muscle fibers, leaving the lipid fractions exposed to free radicals (12). Therefore, oxidation is higher in burgers due to the processing steps, where the grinding of the meat increases the surface area and the incorporation of oxygen and then, the release of enzymes that cause oxidation (65). Enzymatic oxidation occurs from the activity of the enzyme lipases and phospholipases, which are organic catalysts activated when cell membranes are broken in processing or under certain conditions of temperature, humidity, light and oxygen exposure (66). Enzymes catalyze the hydrolytic and oxidative decomposition of fats, which consequently generates numerous volatile compounds that alter the aroma of meat products (67). In this study, this could have contributed to the results obtained in the sensory analysis.

### Sensory analysis

Animals fed with cottonseed grains produced burgers with lower sensory acceptance, regarding to flavor, aroma, and overall quality. This reduction could be due to the amount of grains included in the animals' diet (25.23%). According to Costa et al. (68), diets with 27.51% DM of cottonseed negatively affected the flavor of the meat. Monego et al. (69) showed lower acceptance of burgers when lambs consumed more than 16% cottonseed.

The reduction in consumer acceptance may be indicative of the sensory changes caused by lipid oxidation in the flavor and aroma of the burgers. The flavor and odor of the meat are closely linked, so the evaluation of flavor can be affected by smell (70). In our study, this relationship can be observed by the similar behavior of the means attributed by the judges in the sensory analysis of burger produced with cottonseed.

The juiciness of the burgers decreased after 60 days of storage in our study. The reduction in juiciness over the time of storage may be related to dehydration of the surface exposed to the cold, since the polyethylene packaging does not act as a barrier to environmental conditions. This causes a burn by the freezer that can alter the meat, giving a characteristic rancid flavor (71).

Despite these results, the storage period for burgers is in accordance with the recommendation of AMSA and Food Safety (72) for ground meat and burgers, respectively.

In conclusion, sunflower and soybean grains provide a desirable hypocholesterolemic/hypercholesterolemic ratio and do not negatively influence the concentrations of *n*−3 fatty acids that are essential for human nutrition. We recommend 30 days of storage for burgers made with beef from steers fed soybean and sunflower. The use of diets with cottonseed decreases the sensory acceptance of burgers by consumers, as well as flavor and odor notes.

## Data availability statement

The original contributions presented in the study are included in the article/supplementary material, further inquiries can be directed to the corresponding author/s.

## Author contributions

TB was responsible for preparation of burgers, laboratorial analysis, data analysis and interpretation, and drafting of the manuscript. HV was responsible for the conduction and care of the animals during the feedlot. LÍ was responsible for the conception of study, statistical analysis, and manuscript editing. MD was responsible for acquisition of data and data analysis. MP was responsible for data interpretation and manuscript revision. SC was responsible for acquisition of data. LM for responsible for conception of study and revision of the manuscript. TA was responsible for statistical analysis and manuscript revision. CÍ and RG was responsible for the conception of study. MG was responsible for the conception of the study, supervised data collection, and manuscript writing and revisions. All authors contributed to the article and approved the submitted version.

## Funding

We thank the Coordenação de Aperfeiçoamento de Pessoal de Nível Superior (CAPES) (financial code 001), by financial support, Conselho Nacional de Desenvolvimento Científico e Tecnológico (CNPq), Fundação de Apoio ao Desenvolvimento do Ensino, Ciência e Tecnologia do Estado de Mato Grosso do Sul (FUNDECT), and Federal University of Mato Grosso do Sul that enabled this study to be carried out.

## Conflict of interest

The authors declare that the research was conducted in the absence of any commercial or financial relationships that could be construed as a potential conflict of interest.

## Publisher's note

All claims expressed in this article are solely those of the authors and do not necessarily represent those of their affiliated organizations, or those of the publisher, the editors and the reviewers. Any product that may be evaluated in this article, or claim that may be made by its manufacturer, is not guaranteed or endorsed by the publisher.
